# Size Effects of Copper(I) Oxide Nanospheres on Their Morphology on Copper Thin Films under Near-Infrared Femtosecond Laser Irradiation

**DOI:** 10.3390/nano14191584

**Published:** 2024-09-30

**Authors:** Mizue Mizoshiri, Thuan Duc Tran, Kien Vu Trung Nguyen

**Affiliations:** Department of Mechanical Engineering, Nagaoka University of Technology, Nagaoka 940-2188, Japan

**Keywords:** femtosecond laser reduction, sintering, melting, patterning, copper (I) oxide nanoparticles, nanosphere size

## Abstract

The femtosecond laser direct writing of metals has gained significant attention for micro/nanostructuring. Copper (I) oxide nanospheres (NSs), a promising material for multi-photon metallization, can be reduced to copper (Cu) and sintered through near-infrared femtosecond laser pulse irradiation. In this study, we investigated the size effect of copper (I) oxide nanospheres on their morphology when coated on Cu thin films and irradiated by near-infrared femtosecond laser pulses. Three Cu_2_O NS inks were prepared, consisting of small (φ100 nm), large (φ200 nm), and a mixture of φ100 nm and φ200 nm NSs. A unique phenomenon was observed at low laser pulse energy: both sizes of NSs bonded as single layers when the mixed NSs were used. At higher pulse energies, the small NSs melted readily compared to the large NSs. In comparisons between the large and mixed NSs, some large NSs remained intact, suggesting that the morphology of the NSs can be controlled by varying the concentration of different-sized NSs. Considering the simulation results indicating that the electromagnetic fields between large and small NSs are nearly identical, this differential morphology is likely attributed to the differences in the heat capacity of the NSs.

## 1. Introduction

Laser direct writing of metals has emerged as a promising technique for micro/nanostructuring, with applications spanning electronics, photonics, and plasmonics. Recent advancements have demonstrated its utility in various areas, including electroconductive wiring [1,2,3,4,5,6,7,8], photonic devices [7,9], surface-enhanced Raman spectroscopy (SERS) substrates [10,11,12,13,14,15,16], thermoelectric sensors [17,18,19], and terahertz antennas [20,21,22,23]. Laser-induced metal deposition, a key process in direct writing, has been extensively studied and categorized, particularly in the context of photochemical and thermochemical reactions [24]. Among these, photochemical reactions offer the advantage of finer pattern formation, as the precipitation area is determined by photon density. Femtosecond laser pulse-induced photochemical precipitation of metal is especially noteworthy, as it enables three-dimensional laser direct writing using femtosecond laser pulse-induced photochemical precipitation of metals and achieves three-dimensional microfabrication with a resolution beyond the diffraction limit [14,25,26,27,28,29,30]. To date, this technique has been employed to form microstructures of gold, silver, and Mo–Co–W alloys through multi-photon excitation and subsequent precipitation [1,3,4,6,25,26,27,28,29,30]. However, the use of this method has been largely limited to noble metals and specific alloys, reducing its versatility.

Thermochemical reactions, on the other hand, have broadened the scope of laser direct writing to a wider variety of metals. The excitation process in thermochemical reactions can be classified into single- and multi-photon excitations. In single-photon excitation, the raw materials exhibit strong absorption at the laser wavelength. For example, copper (II) oxide nanoparticles (CuO) with a band gap of 1.2 eV can be reduced and sintered to form copper (Cu) patterns through a thermochemical reaction when irradiated with continuous-wave and nanosecond lasers with the wavelength of 1070 nm. This process uses CuO nanoparticles mixed with ethylene glycol as a reducing agent and polyvinylpyrrolidone (PVP) as a dispersant [31]. Near-infrared and green femtosecond laser pulses have also been utilized for the thermochemical reduction of CuO nanoparticles, resulting in the formation of Cu, Cu_2_O, and CuO patterns [9,32,33,34,35,36]. The concentration of these patterns can be controlled by adjusting the laser irradiation parameters, such as the laser writing speed and pulse energy [32]. Laser-reductive sintering has been applied to metals like nickel, cobalt, copper–nickel alloys, using their respective oxide nanoparticles [17,37,38,39,40,41,42].

In multi-photon excitation followed by thermochemical reactions, the absorption properties of the raw material inks and the wavelength of femtosecond lasers are crucial. The inks must exhibit high transmittance at the laser wavelength and strong absorption at wavelengths below half the laser wavelength. Glyoxylic acid (GA)–metal complex solutions such as those for Cu and Ni have been synthesized as inks, enabling the formation of high-purity metal and alloy patterns [43]. However, those solution-based inks are limited in their ability to form thick layers in a single laser scan due to their low metal ion density [43,44,45,46,47,48,49].

Solid inks, such as nanoparticles, offer an advantage by minimizing shrinkage from the raw ink to the precipitated metal. Copper (I) oxide (Cu_2_O) nanoparticles, with a band gap of 2.1 eV, are particularly promising for multi-photon excitation followed by thermochemical metallization. Cu_2_O nanoparticles exhibit high transmittance at femtosecond laser wavelengths of 780 nm and 515 nm, with intense absorption below 400 nm [50,51,52]. Spherical Cu_2_O nanoparticles, or Cu_2_O nanospheres (NSs), have been synthesized using the polyol method [53] and mixed with 2-plopanol and PVP to create Cu_2_O NS inks. These inks, prepared with various NS sizes, have been studied for their printing properties. While both small (φ~100 nm) and large (φ~200 nm) Cu_2_O NSs can be used for Cu patterning, smaller NSs (φ~100 nm) result in higher conductive patterns.

During patterning on different substrates, a unique single-NS bonding phenomenon was observed on Cu thin films [51]. This bonding was likely due to the electromagnetic field between semiconductor Cu_2_O NSs and the Cu thin film at the contact point, leading to localized heating and bonding. The single-layered Cu_2_O bonding on Cu thin films suggests potential applications in three-dimensional microfabrication, where underlying Cu in the Cu_2_O NSs induces localized heating, reduction, and layer-by-layer lamination. Additionally, the bonding states were classified into four groups, such as single-layer bonding, multi-layer bonding, melting, and the formation of a laser-induced periodic surface structure (LIPSS). However, the size effect of Cu_2_O NSs on their morphologies on Cu thin films under femtosecond laser pulse irradiation remains unclear.

In this study, we investigate the morphology of patterns formed using Cu_2_O NSs with large (~200 nm) and small (~100 nm) diameters. By using inks containing large, small, and mixed Cu_2_O NSs, we aim to understand the effect of laser irradiation on morphology. We experimentally evaluate the size dependence of the bonding states under varying laser irradiation conditions, such as pulse energy, and perform an electromagnetic field analysis to explore the bonding mechanism.

## 2. Materials and Methods

### 2.1. Preparation of Cu_2_O NS Inks

Cu_2_O NSs were synthesized using a polyol method. We have previously reported the synthesis of large and small Cu_2_O NSs by controlling the reaction conditions [50,51,52]. To investigate the size effect of Cu_2_O NSs on direct writing on Cu thin films, three Cu_2_O NS inks with different particle distributions were prepared, with one containing only φ100 nm, one containing only φ200 nm, and one containing a mixture of φ100 nm and φ200 nm NSs. The ratio of φ100 nm and φ200 nm NSs in the mixed NS ink was set at 1:2 wt% to achieve the calculated geometric filling rate of over 90%, compared to the approximately 74% when using monodispersed NSs. Both sizes of NSs were prepared using the polyol method [53]. The concentrations of Cu_2_O NSs, 2-propanol, and PVP (M_w_~55,000) in the ink were 1.70 wt%, 95.25 wt%, and 3.05 wt%, respectively.

### 2.2. Femtosecond Laser Irradiation of Cu_2_O NS Ink

**Figure 1:**
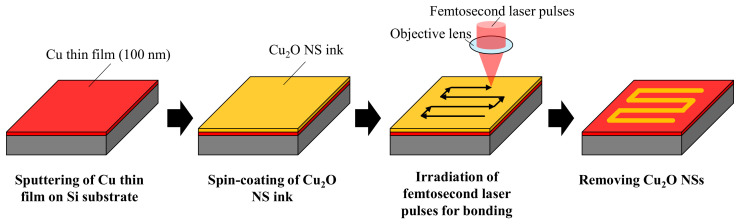
A schematic illustration of the direct writing process using femtosecond laser pulse-induced reductive sintering and/or melting of Cu_2_O NSs.

Figure 1 shows a schematic illustration of the direct writing process using femtosecond laser pulse-induced reductive sintering and/or melting of Cu_2_O NSs. First, the Cu thin films with a thickness of approximately 100 nm were deposited on Si substrates (thickness: 525 µm ± 25 µm) using radio-frequency (RF) magnetron sputtering (Canon ANELVA, L-250S-FH, Tokyo, Japan). The RF power, argon gas operating pressure, and deposition duration were set at 30 W, 1 Pa, and 5 min, respectively.

Next, the Cu_2_O NS ink was spin-coated on the Cu thin films using a spin-coater (MIKASA, MS-A100, Tokyo, Japan). The spin-coating process involved spinning at 500 rpm for 10 s, followed by baking at 80 °C on a hot plate for 8 min to solidify the Cu_2_O NS ink films. Linearly polarized femtosecond laser pulses with a wavelength of 780 nm, pulse duration of 120 fs, and repetition frequency of 80 MHz were then focused onto the surface of the inks using an objective lens with a numerical aperture of 0.80. The spot diameter was approximately 1 µm, measured by the knife-edge method. Cu_2_O NS inks are already known for their excellent nonlinear optical absorption against near-infrared femtosecond laser pulses with a wavelength of 780 nm [50,51,52]. The patterns were written by scanning the substrates using an xyz-mechanical stage (ALIO Industries, Inc., AI-CM-60, Arvada, CO, USA). The writing speed was set at 0.1 mm/s, as the single-layer bonding of the Cu_2_O NSs was obtained at this low writing speed in previous studies [24]. Finally, the sample substrates were rinsed in ethanol to remove the non-sintered or non-melted Cu_2_O NSs.

### 2.3. Evaluation Methods

The morphology of the Cu_2_O NSs was observed using field-emission-scanning electron microscopy (FE-SEM, SU-8200, Hitachi High-Tech Corporation, Tokyo, Japan). The particle size distribution was analyzed using ImageJ (https://imagej.net/ij/), an image processing software. For observation, the Cu_2_O NSs were dispersed in a H_2_O and ethanol mixed solution (1:1), coated on glass substrates, and dried at 80 °C, as described in previous reports [50,51,52]. The absorption coefficient of the inks was measured using an ultraviolet–visible spectrophotometer (Shimadzu, UV-2600, Kyoto, Japan). The morphology and line width of the patterns written directly on the substrates were also observed using FE-SEM.

The electromagnetic fields generated during the laser irradiation of the Cu_2_O NSs on the Cu thin films were calculated using the finite element method (FEM) with COMSOL Inc., COMSOL Multiphysics (v6.2, Burlington, MA, USA). To account for the effect of linear polarization of the laser pulse on the electromagnetic fields between the NSs and the Cu thin films, three-dimensional models consisting of two layers of Cu_2_O NSs on the Cu thin film were used. To compare the electromagnetic field between the Cu_2_O NSs and the underlying Cu thin film, first, the electromagnetic field was calculated using the FEM. Then, the absorption intensity was also calculated using the intensity of the electromagnetic field and the absorption coefficients of the Cu_2_O NSs and Cu thin film. Here, the scattering effect was assumed to be negligible.

## 3. Results and Discussion

### 3.1. Preparation of Cu_2_O NS Inks Using Different Sizes of Cu_2_O NSs

**Figure 2:**
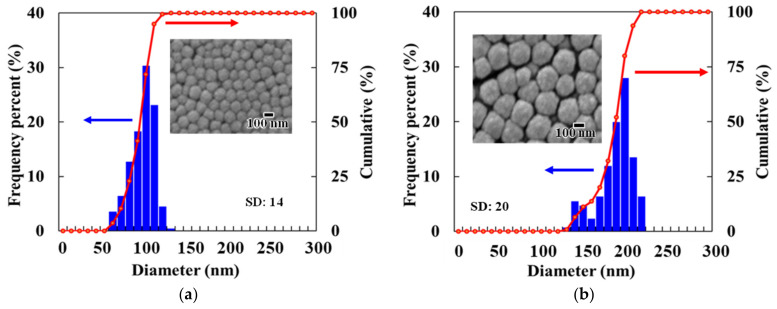
FE-SEM images and particle size distributions of Cu_2_O NSs with diameters of (**a**) 100 nm and (**b**) 200 nm.

Figure 2 shows the FE-SEM images and particle size distributions of the Cu_2_O NSs. The NSs prepared under the conditions specified in Table 1 exhibited the diameters of approximately 100 nm and 200 nm, with standard deviations of 14 and 20, respectively. These results indicate that the NSs are well monodispersed.

**Figure 3:**
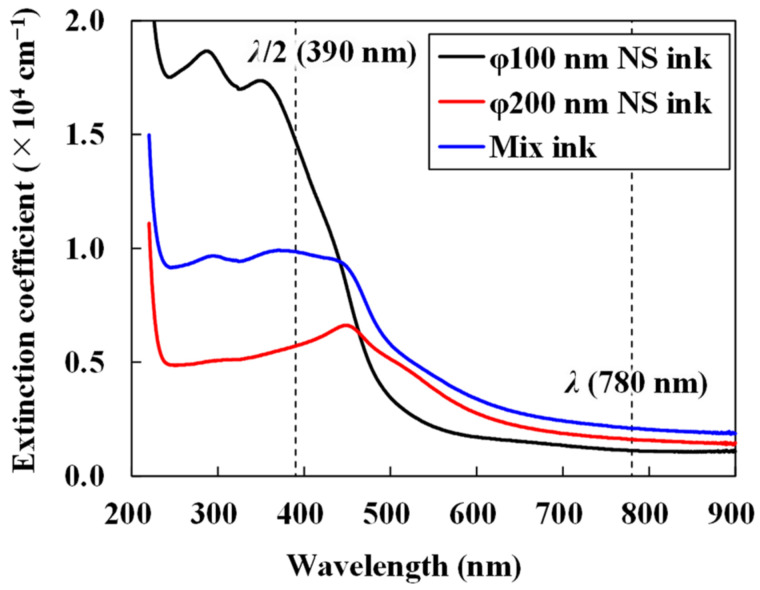
Absorption coefficients of three Cu_2_O NS inks.

Figure 3 presents the absorption coefficients of the three Cu_2_O NS inks containing only φ100 nm NSs, φ200 nm NSs, and a mixture of φ100 nm and φ200 nm NSs. All the inks demonstrated high transmittance at the laser wavelength of 780 nm and intense absorption at half the wavelength. These findings are consistent with previous reports on both φ100 nm and φ200 nm NSs [52]. Here, the absorption strictly included both absorption and scattering by the Cu_2_O NSs because the refractive indices of the Cu_2_O NSs, PVP, and 2-propanol were approximately 2.262 + *i*0.025, 1.53, and 1.37, respectively [51]. Considering the wavelength and the size of the Cu_2_O NSs, Rayleigh scattering was possibly caused in the Cu_2_O NS inks when the laser pulses were irradiated. However, the thickness of the Cu_2_O NS inks was approximately 0.5 µm, which indicated that the number of Cu_2_O NSs were maximally approximately five. Therefore, the scattering was assumed to be negligible. Consequently, these three Cu_2_O NS inks were used to investigate the particle size effect on femtosecond laser-reductive sintering/melting of the Cu thin films.

### 3.2. Patterning Properties

**Figure 4:**
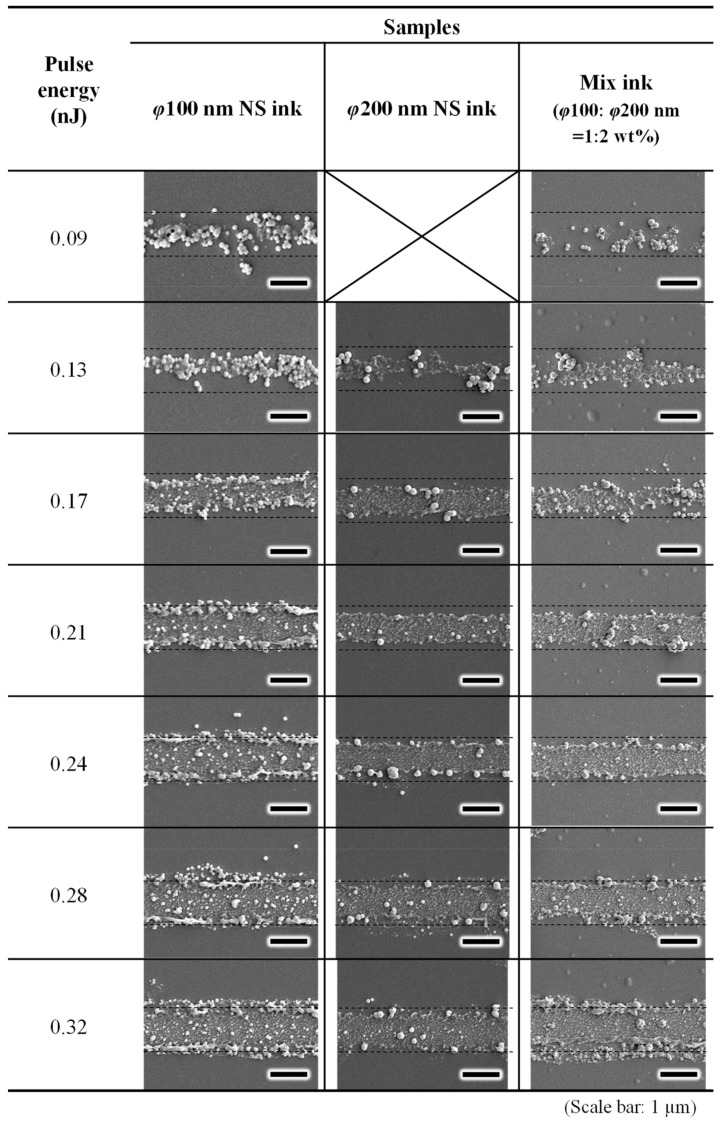
FE-SEM images of line patterns fabricated on Cu thin film-coated Si at various laser pulse energies with a writing speed of 0.1 mm/s.

**Figure 5:**
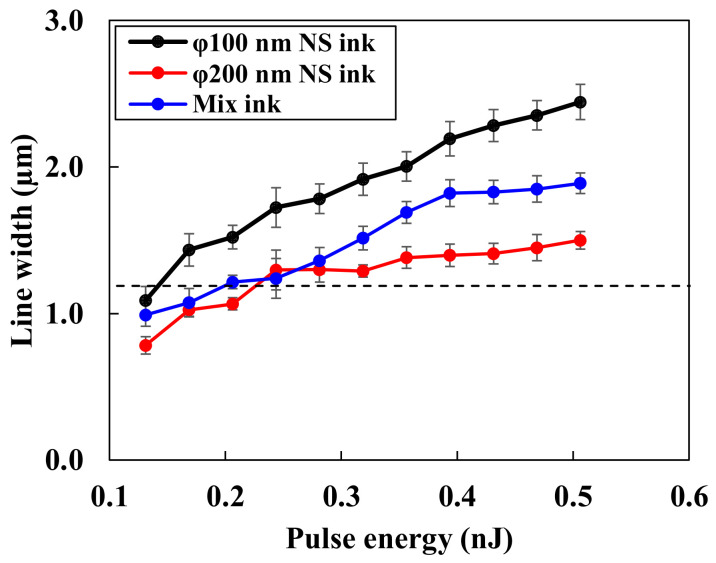
Relationship between line width and laser pulse energy.

To evaluate the size effect of the Cu_2_O NSs on their morphologies on the Cu thin films irradiated by near-infrared femtosecond laser pulses, three inks consisting of large, small, and mixed Cu_2_O NSs were irradiated with varying laser pulse energies. Figure 4 shows the SEM images of the line patterns. The dashed line in the images indicates the scanning area of the laser pulses. Figure 5 depicts the relationship between the pulse energy and line width. Both the large and mixed NSs produced a line width finer than the focal spot diameter. This suggests that the direct writing phenomenon is dependent on the laser pulse threshold rather than thermal diffusion, which is consistent with previous findings on Cu_2_O NS bonding on Cu thin films [51]. The line width could not be recognized at a pulse energy of 0.09 nJ. Especially, the Cu_2_O NSs with φ200 nm were not bonded to the substrate after rinsing the sample, although the small NSs were partially bonded to the substrates. Considering that the heat capacities of the Cu_2_O NSs with φ200 were 8 times larger than those with φ100 nm, these results suggest that the small NSs were heated sufficiently for bonding and melting to remain after rinsing the samples.

**Figure 6:**
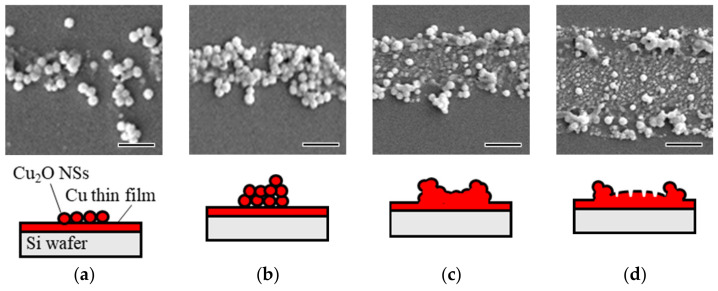
SEM images of the typical four bonding states on the Cu thin film-coated Si substrates: (**a**) single-layered, (**b**) multi-layered, (**c**) melted NSs, and (**d**) LIPSS. Scale bar: 500 nm.

The morphological states of the line patterns were classified into four categories: single-layered, multi-layered, melted, and LIPSS. Table 2 summarizes the morphological classifications based on the pulse energy. Figure 6 shows the typical four bonding states on the Cu thin film-coated Si substrates: single-layered, multi-layered, melted NSs, and LIPSS. These classifications followed the research previously reported [51]. For the single-layered bonding, it was mentioned that the localized plasmon resonance enhanced the electromagnetic field between the Cu_2_O NSs and the underlying Cu thin film in the TM mode only, indicating that the localized heating and bonding were induced. As the pulse energy increased, the Cu_2_O NSs in all the inks melted and formed an LIPSS, indicating that higher pulse energies led to excessive irradiation.

A unique phenomenon was observed where both sizes of NSs bonded as single layers when using the mixture NSs at low laser pulse energies. At higher pulse energies, the small NSs melted more readily than the large NSs. In comparison, some large NSs remained intact, suggesting that the morphology of NSs can be controlled by varying the concentration of different sizes of NSs.

### 3.3. Effect of Cu Thin Films on Electromagnetic Field under Irradiating a Femtosecond Laser Pulse

**Figure 7:**
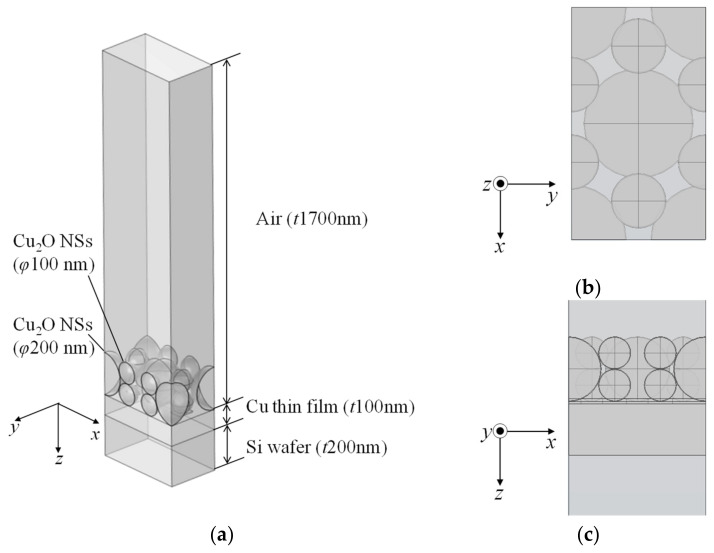
(**a**) Three-dimensional overall view of Cu_2_O NS mixed model, (**b**) xy-plane view, and (**c**) xz-plane view for calculating electromagnetic fields.

The electromagnetic fields generated during femtosecond laser irradiation were calculated using the FEM. Figure 7 illustrates the model for the calculations. The Cu_2_O NSs with diameters of 100 nm and 200 nm were densely aligned. The Cu thin film thickness was set to 100 nm, consistent with the experimental conditions. The thicknesses of the Si substrate and air layers were chosen to be at a minimum so that their effects were negligible. Periodic boundary conditions were applied on the xz plane and yz plane, while the xy planes at the ends of the air and Si layers were assigned scattering and perfect electric conductor conditions, respectively, to prevent unexpected reflections. The contact points between the Cu_2_O NSs and Cu_2_O/Cu interface were meshed with sizes less than 1 nm. The applied electromagnetic field was defined with an amplitude of 1 and linearly polarized. The material properties used in the FEM are shown in Table 3.

**Figure 8:**
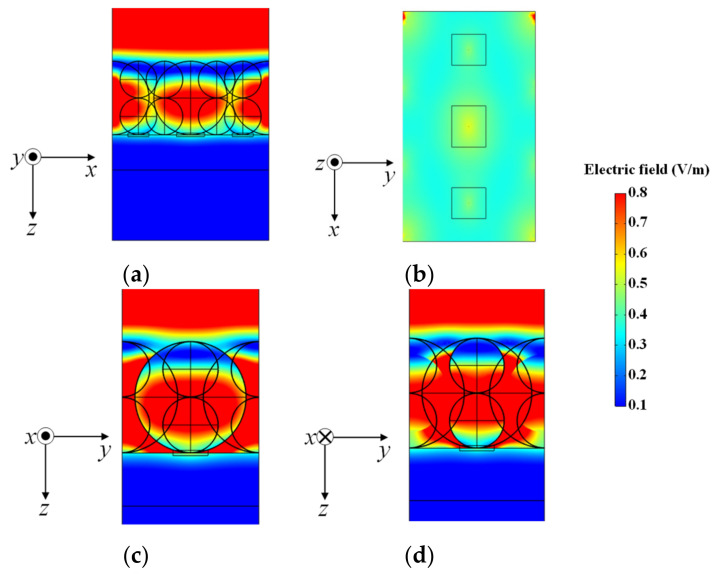
Electric field distribution for the φ200 nm/φ100 nm Cu_2_O NS mix model: (**a**) xz plane, (**b**) yz plane (φ200 nm NS side), (**c**) yz plane (φ100 nm NS side), and (**d**) xy plane of the contact point between the Cu_2_O NS and Cu thin film.

First, the norm of the electric field was calculated under the applied electromagnetic field of femtosecond laser pulses. Figure 8 shows the electric field distribution. While there was no significant enhancement at the contact points between the Cu_2_ONSs, except for some interference effects, notable enhancement occurred at the contact points between the Cu_2_O NSs and the Cu thin film, as shown in Figure 8b.

**Figure 9:**
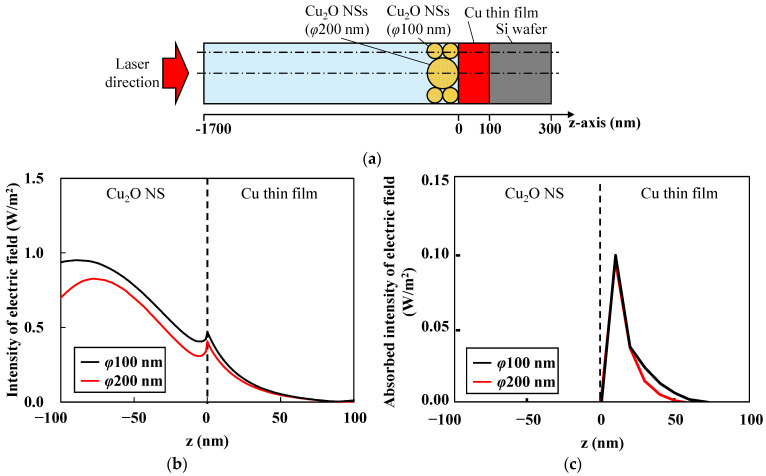
(**a**) Schematic illustration of the cross-sectional model showing the *z*-axis penetrating the φ200 nm and φ100 nm Cu_2_O NSs. (**b**) Electric field intensity distribution along the *z*-axis, and (**c**) absorbed electric field intensity distribution along the *z*-axis.

Next, the intensity of the electromagnetic fields absorbed by each material was evaluated. The absorbed intensity *I*_abs_ was calculated as the product of the electric field intensity and the absorption coefficient. The electric field intensity is defined by the square of the norm of the electromagnetic field. Figure 9a,b illustrate the intensity of the electric field and the absorbed intensity of the electric field along the *z*-axis, including the contact point between the Cu_2_O NSs and Cu thin films. These results suggest that the size of the Cu_2_O NSs did not significantly affect the intensities between the Cu_2_O NSs and Cu thin films. Given that the electromagnetic fields between the large and small NSs were nearly identical, the observed differences in morphology are attributed to the heat capacity of the NSs. Considering the specific heat capacity *C*_p_ 568 J/(kg K), volume *V* (1.25 × 10^−16^ cm^3^), and density *ρ* (6000 kg/cm^3^) of Cu_2_O, the heat capacity was estimated to be 1.78 nJ/K (*ρVC*_p_) when using Cu_2_O NSs with a 100 nm diameter. When using Cu_2_O NSs with a 200 nm diameter, the heat capacity was 8 times larger than that of Cu_2_O NSs with a 100 nm diameter. The temperature will be estimated by considering the heat accumulation using the heat transfer analysis.

## 4. Conclusions

In this study, we investigated the effects of Cu_2_O NSs of different sizes on the morphology of patterns formed on copper thin films under near-infrared femtosecond laser irradiation.

(1) Cu_2_O NSs with diameters of ~100 nm and ~200 nm were successfully synthesized and used to prepare inks. The patterning experiments revealed that the morphology of the laser-written patterns was significantly influenced by the size of the NSs. At lower laser pulse energies, NSs of both sizes bonded as single layers when using the mixed ink. However, at higher pulse energies, smaller NSs melted more readily compared to larger NSs, resulting in differences in the final morphology of the patterns.
(2) The morphology of the patterns was categorized into single-layered, multi-layered, melted, and laser-induced periodic surface structures (LIPSSs). As the pulse energy increased, all the NSs melted and formed LIPSSs, indicating that high pulse energies led to excessive irradiation. The results suggest that the concentration of different sizes of NSs can be used to control the morphology of the patterns by adjusting the laser pulse energy.
(3) Finite element method (FEM) simulations showed that the electromagnetic field intensities at the contact points between the NSs and the Cu thin films were similar for both small and large NSs. This finding implies that the observed differences in morphology are not due to variations in the electromagnetic field intensity but rather due to the heat capacity of the NSs. The simulations confirmed that the size-dependent morphological effects were influenced by the thermal properties of the NSs rather than by differences in the electromagnetic field distribution.

Overall, both the experimental and simulation results highlight that the direct writing properties of Cu_2_O NSs using femtosecond laser-reductive sintering are influenced by the underlying substrate as well as the initial absorption characteristics. Additionally, in the case of thick Cu_2_O NS ink films used for three-dimensional structuring, extinction due to scattering may influence the internal writing properties. This scattering loss can be mitigated by optimizing the refractive indices between the Cu_2_O NSs and the surrounding reducing agents. Using a high-refractive-index solution, such as a mixture of ethylene glycol (1.43) and PVP (~1.53), and varying the sizes of the Cu_2_O NSs can reduce these losses. These insights are valuable for optimizing layer-by-layer microfabrication techniques for three-dimensional structures.

## Figures and Tables

**Table 1:** Synthesis conditions for Cu_2_O NSs.

Size	Cu(NO_3_)_2_·2.5H_2_O (g)	Ethylene Glycol (mL)	PVP (g)	Injecting Speed (mL/h)	Oil Bath Temperature (°C)
φ100	0.20	44	0.39	50	160
φ200	0.20	44	1.56	100	140

**Table 2:** Relationship between pulse energy and bonding states with different inks.

Pulse Energy (nJ)	φ100 nm NS Ink	φ200 nm NS Ink	Mixed Ink (φ100 nm: φ200 nm = 1:2 (wt%))
0.09	**S** and Melted	Non-bonded	**S**
0.13	**M** and Melted	**S** and Melted	**M** and Melted
0.17	**M** and Melted	**S** and Melted	**M** and Melted
0.21	Melted and LIPSS	**S** and Melted	**M** and Melted
0.24	Melted and LIPSS	**S** and Melted	Melted and LIPSS
0.28	Melted and LIPSS	Melted and LIPSS	Melted and LIPSS
0.32	Melted and LIPSS	Melted and LIPSS	Melted and LIPSS

**S**: single-layered, **M**: multi-layered, and LIPSS: laser-induced periodic surface structure.

**Table 3:** Material properties used in FEM.

Materials	Refractive Index *n*	Extinction Coefficient *k*	Absorption Coefficient (cm^−1^)
Cu_2_O NSs	2.262	0.025	4027
Cu thin film	0.247	4.855	782,100
Si substrate	3.71	0.0077	1240
Air	1	0.001	0

## Data Availability

The data will be made available on request.
